# Automated data extraction for systematic reviews using GPT‐5.2 and Google Gemini Pro 3: A dual‐large language model approach in orthopaedic research

**DOI:** 10.1002/ksa.70412

**Published:** 2026-04-21

**Authors:** Prushoth Vivekanantha, Harjind Kahlon, Oluwatoba T. Balogun, Marc Daniel Bouchard, Darren de Sa, Olufemi R. Ayeni, Jeffrey Kay

**Affiliations:** ^1^ Division of Orthopedic Surgery, Department of Surgery McMaster University Hamilton Ontario Canada; ^2^ Temerty School of Medicine University of Toronto Toronto Ontario Canada

**Keywords:** artificial intelligence, automation, extraction, large language model, systematic review

## Abstract

**Purpose:**

To evaluate the accuracy, agreement, and efficiency of a dual‐large language model (LLM) approach using Generative Pre‐Trained Transformer 5.2 (GPT‐5.2) and Google Gemini 3 Pro for automated data extraction in orthopaedic systematic reviews.

**Methods:**

Eight studies from a previously published systematic review on paediatric revision anterior cruciate ligament reconstruction were used to test extraction accuracy, agreement, and efficiency against a pre‐defined gold‐standard. Both GPT 5.2 and Gemini 3 Pro were prompted via the OpenAI and Google Application Programming Interface (API). Each study had a total of 48 equally weighted data fields to extract from spanning six domains: study characteristics, participant details, injury characteristics, primary and revision surgery details, and outcomes. Extractions were graded as correct, partially correct, or incorrect in reference to the gold‐standard.

**Results:**

Across all 384 fields, both LLMs produced fully correct outputs in 315 (82%) cases, while at least one model was fully correct in 365 (95.1%). Among the six extraction domains, study characteristics (100%, 32/32), injury characteristics (93.8%, 30/32), and outcomes (91.1%, 102/112) showed the highest percentage of at least one model being correct. The entire extraction task was completed in 27 and 35.8 min by GPT‐5.2 and Gemini 3 Pro, respectively, for a total API cost of $3.22USD.

**Conclusion:**

A parallel‐LLM approach using GPT‐5.2 and Gemini 3 Pro achieved strong accuracy with a high degree of efficiency for automated data extraction in an orthopaedic systematic review. Most errors were due to omission of minor details in complex domains such as surgical details. At least one model was fully correct in over 95% of fields, supporting the use of a dual‐LLM framework as a reliable first‐pass tool for human verification.

**Level of Evidence:**

Level IV.

**Abbreviations**
ACL: anterior cruciate ligament
ACLR: anterior cruciate ligament reconstruction
AI: artificial intelligence
API: application programming interface
GPT: Generative Pre‐Trained Transformer
IKDC: International Knee Documentation Committee
IQR: interquartile range
LLM: large language model
NLP: natural language processing
PDF: portable document format
Pedi‐IKDC: Paediatric International Knee Documentation Committee
RCT: randomised controlled trial
SD: standard deviation
USD: United States Dollars

## INTRODUCTION

Systematic reviews are key contributors to evidence‐based orthopaedics, informing clinical guidelines and surgical decision‐making across all subspecialties [2, 5, 9, 18]. The process of completing a high‐quality systematic review is time‐consuming and resource‐intensive [6]. Data extraction represents a phase of the systematic review workflow that requires a significant amount of manual work and is highly prone to errors [30]. Extraction requires the interpretation of several studies with often heterogeneous reporting and methodological formats [7]. Despite the standard practice of duplicate extraction followed by conflict‐resolution and adjudication, missed or wrong data entries may persist into the final analysis [29, 30].

Artificial intelligence (AI), specifically, large language models (LLMs), have emerged as potential tools for automating the phases of the systematic review workflow, including citation screening [11, 20, 27]. Preliminary studies have outlined that LLMs can dramatically reduce the workload experienced by authors and accelerate the synthesis of these studies, performing similar to humans in accuracy [11, 20, 27]. Specifically, recent studies have investigated the role of LLMs for extraction, suggesting that these models can expedite this task with a moderate to strong performance [16, 17, 24]. Extraction tasks are methodologically and technically different from screening, with separate challenges.

One potential strategy to improve the reliability of LLMs is the use of a parallel model approach, similar to that of human extraction. Rather than solely relying on one LLM, two independent LLMs can extract data from the same study, with disagreements and conflicts that arise used to identify data that require human verification. Different LLMs may exhibit different yet complementary strengths, thus pairing models may enhance performance. This strategy has seldom been investigated in the literature, especially within the field of orthopaedic surgery. Therefore, the objective of this study was to evaluate a parallel LLM extraction framework using Generative Pre‐Trained Transformer 5.2 (GPT‐5.2) and Google Gemini 3.0 Pro for automated extraction of variables from orthopaedic studies. Specifically, extraction accuracy relative to a human reference standard, inter‐model agreement, and efficiency metrics were evaluated. It is hypothesised that a dual‐large language model will have a high‐degree of efficacy and efficiency in automated data extraction for systematic reviews.

## METHODS

### Study selection

Studies from a previously published orthopaedic systematic review from the study group was used to test the extraction performance of GPT‐5.2 and Google Gemini 3.0 Pro [26]. The purpose of the systematic review was to outline the surgical techniques and clinical outcomes in paediatric and adolescent patients undergoing revision anterior cruciate ligament reconstruction (ACLR) [26]. This systematic review synthesised data from eight different primary studies including a wide variety of patient demographics, injury details, surgical techniques, and clinical outcomes. This topic was chosen because paediatric revision ACLR involves complex surgical reporting, a wide variety of outcome measures, and detailed operative variables, making it a difficult test case for automated extraction. The extraction schema was developed a priori based on the original systematic review protocol prior to LLM testing.

### Data elements for extraction

An extraction schema was developed a priori and was broken down into the following domains: study identification, participant characteristics, injury characteristics, primary surgery details, secondary or revision surgery details and clinical outcomes.

Study identification variables included first author, year of publication, journal of publication, and study design with level of evidence. Participant characteristics included the final number of patients, final number of knees, follow‐up duration, lost to follow‐up, sex distribution, age at primary and revision surgery, time interval between primary surgery and failure, time interval between primary surgery and revision and physeal status at both primary and revision procedures.

Injury characteristics included both mechanisms of injury at both primary and secondary injuries as well as the main sports of patients in both the primary and secondary injury settings. Primary surgery details included graft choice, graft diameter, tunnel drilling technique, femoral and tibial fixation methods, and concomitant injuries and procedures. Similar variables as that of primary surgery details were extracted for the revision setting, however an additional variable was added, specifically the use of new versus reused tunnels. Outcomes included partial and complete graft failures, total failures, time to revision graft failure, contralateral ACL injuries, postoperative Lachman and pivot‐shift grades, return to sport both in general and at the same level, time to return to sport, and patient reported outcome measures such as the paediatric International Knee Documentation Committee (Pedi‐IKDC), IKDC, Lysholm and Tegner scales. All domains and data fields were weighted equally.

For all continuous variables, all central tendencies (e.g., mean, median), and all variances (standard deviations [SD], interquartile range [IQR] and ranges) were required to be reported in order to be considered complete and accurate. For all variables requiring counts, both the total number and percentage were required to be reported to be considered complete and accurate. A full list of extraction variables with associated rules can be found in Appendix: Table A1.

### Human extraction and development of gold‐standard reference

Two medical students (HK and OB) independently performed manual data extraction from all included primary studies using a predefined extraction schema. Reviewers worked in parallel and were blinded to each other's extractions. Following the independent extraction process, the two medical students underwent conflict resolution to create a consolidated file of extracted variables. After this, an orthopaedic surgery resident (PV) served as a third reviewer to independently verify the accuracy of the consolidated extractions. This final dataset was considered the human gold‐standard extraction used for comparison with the LLMs. This framework was chosen as it is standard for systematic reviews to have junior learners such as medical students perform data extraction followed by verification by a more senior author such as an experienced orthopaedic resident.

### Large language model extraction workflow

For each of the eight studies, portable document formats (pdfs) of eligible manuscripts were downloaded and organised into a folder. LLM extraction was performed using the application programming interfaces (API) of Google (Mountainview, California, United States) and OpenAI (San Francisco, California, United States), calling on the Gemini 3.0 Pro and GPT‐5.2 model (thinking mode: high), respectively. Custom natural language processing (NLP) python scripts were used for both LLMs utilising an identical prompt with the same extraction instructions. Extraction was performed independently, and models did not have access to the other's outputs. Python scripts were created a priori. Model temperature and randomness parameters were fixed to ensure reproducibility. The prompt utilised for both models can be found in Appendix: Table A2.

### Classification of extraction accuracy

Each of the extracted data generated by both LLMs were independently evaluated against the human gold standard by the orthopaedic resident reviewer (PV) and classified as either correct, partially correct, or incorrect. An extraction was deemed to be correct if the model output exactly matched the gold‐standard value or was semantically equivalent without the loss or manipulation of information. This included: identical or equivalent reporting of central tendencies, variance measures and units for continuous variables, correct and complete reporting of counts and percentages for non‐continuous outcomes, identical or equivalent reporting of string or text‐based variables, and accurate reporting of 'not reported' when indicated.

An extraction was considered partially correct if the model contained some accurate information of the variable but was incomplete or imprecise against the human gold‐standard. Examples of this include: (a) a correct central value but missing or incomplete reporting of variances (e.g., correct mean and SD reported but missing range when range was variable), (b) a correct count reported with a lack of percentage reported or vice‐versa and (c) incomplete reporting of a given subgroup (e.g., a model reporting on all possible graft types used in a study with correct counts and percentages but misses one graft option reported in the original study). Incorrect extractions were classified if the model did not match the human gold standard, contained incorrect values, or represented a misinterpretation of the prompt or a hallucination. Partial correctness was kept as a separate category to distinguish real‐world extraction workflows where errors were due to minor omissions rather than complete failures or hallucinations. The orthopaedic resident was not blinded to LLM outputs during verification.

### Parallel model accuracy classification

Paired outputs for GPT‐5.2 and Gemini 3.0 Pro were jointly classified into one of the following categories: (1) GPT and Gemini both correct, (2) GPT and Gemini both incorrect, (3) GPT and Gemini both partially correct, (4) GPT correct but Gemini partially correct, (5) Gemini correct but GPT partially correct, (6) GPT incorrect but Gemini partially correct, (7) Gemini incorrect but GPT partially correct, (8) GPT correct but Gemini incorrect and (9) Gemini correct but GPT incorrect. Percentage agreements were measured across all extraction fields as well as for individual extraction domains (study identification, participant characteristics, injury characteristics, primary surgery details, secondary or revision surgery details and clinical outcomes).

### Statistical analysis

Descriptive statistics were used to summarise extraction accuracy and agreement across all extraction fields and domains. Binomial 95% confidence intervals for accuracy proportions were calculated using the Wald score method for main accuracy scores [1].

#### Inter‐model agreement

Inter‐model agreement was also assessed, evaluating the extent to which GPT‐5.2 and Gemini 3.0 Pro produced the same outcome for a given data extraction variable. Two model outputs were considered in agreement if they were identical or semantically equivalent (complete and identical counts, percentages, central tendencies and variances).

#### Efficiency outcomes

To assess efficiency and feasibility, runtime and API usage cost were tracked. The total time elapsed for extraction was recorded. Similarly, API costs were recorded using OpenAI's and Google's available per‐token pricing for the GPT‐5.2 and Gemini 3 Pro models, respectively. For GPT‐5.2, the equation was as follows: cost = (input tokens × 1.75/1,000,000) + (output tokens × 14/1,000,000). For Gemini 3 Pro, the equation was as follows: cost = (input tokens × 2.0/1,000,000) + (output tokens × 12/1,000,000). A token is the smallest unit of text that the model is able to process or generate.

## RESULTS

### Model performance

Across eight included studies with 48 different variables to extract per study, a total of 384 individual fields were extracted with the parallel LLM extraction framework. Overall, both models produced fully correct extractions in 315 fields (82%, 95% CI 78.2%–85.9%). At the individual study level, the proportion of fields where both models were fully correct ranged between 60.4% and 89.6%. When considering both models together, at least one model generated a fully correct extraction for 365 (95.1%, 95% CI 92.9%–97.2%) fields, ranging between 72.9%–95.8% at the individual study level. Both models were incorrect for 15 (3.9%) total variables, ranging between 0% and 12.5%. Both models generated a partially correct response in 28 (7.3%) total variables, ranging 0%–22.9%.

GPT‐5.2 was fully correct while Gemini 3 Pro was partially correct in only eight fields (2.1%), where Gemini 3 Pro was fully correct with GPT‐5.2 being partially correct in two fields (0.5%). More asymmetric errors were rare, with GPT‐5.2 being correct and Gemini being incorrect in five fields (1.3%) and vice‐versa in nine fields (2.3%). The total number of agreements where both models reported the same response, either partially correct or both corrects was 342 (89.1%). Performance per study can be found in Table 1.

**Table 1:** Large language model performance across included studies.

Outcome category	Christino et al. [3]	Cooper et al. [4]	Ouilette et al. [19]	Reinhardt et al. [21]	Rugg et al. [22]	Saper et al. [23]	Shelbourne et al. [25]	Winkleret al. [28]	Total
Both correct	29 (60.4%)	40 (83.3%)	39 (81.3%)	42 (87.5%)	40 (83.3%)	42 (87.5%)	43 (89.6%)	40 (83.3%)	315 (82.0%)
Both incorrect	0 (0.0%)	6 (12.5%)	2 (4.2%)	2 (4.2%)	1 (2.1%)	1 (2.1%)	1 (2.1%)	2 (4.2%)	15 (3.9%)
Both partially correct	11 (22.9%)	0 (0.0%)	3 (6.3%)	2 (4.2%)	5 (10.4%)	4 (8.3%)	1 (2.1%)	2 (4.2%)	28 (7.3%)
GPT correct/GEMINI partial	6 (12.5%)	0 (0.0%)	1 (2.1%)	0 (0.0%)	0 (0.0%)	0 (0.0%)	1 (2.1%)	0 (0.0%)	8 (2.1%)
GEMINIi correct/GPT partial	0 (0.0%)	0 (0.0%)	1 (2.1%)	0 (0.0%)	0 (0.0%)	0 (0.0%)	1 (2.1%)	0 (0.0%)	2 (0.5%)
GPT incorrect/GEMINI partial	1 (2.1%)	0 (0.0%)	0 (0.0%)	0 (0.0%)	0 (0.0%)	0 (0.0%)	0 (0.0%)	0 (0.0%)	1 (0.3%)
GEMINIincorrect/GPT partial	1 (2.1%)	0 (0.0%)	0 (0.0%)	0 (0.0%)	0 (0.0%)	0 (0.0%)	0 (0.0%)	0 (0.0%)	1 (0.3%)
GPT correct/GEMINI incorrect	0 (0.0%)	1 (2.1%)	0 (0.0%)	1 (2.1%)	1 (2.1%)	1 (2.1%)	0 (0.0%)	1 (2.1%)	5 (1.3%)
GEMINI correct/GPT incorrect	0 (0.0%)	1 (2.1%)	2 (4.2%)	1 (2.1%)	1 (2.1%)	0 (0.0%)	1 (2.1%)	3 (6.3%)	9 (2.3%)

Abbreviations: GEMINI, Gemini 3 Pro; GPT‐5.2, Generative Pre‐Trained Transformer.

### Sensitivity analysis of errors

#### Study characteristics

Across 32 data fields representing four variables extracted from eight studies, both GPT‐5.2 and Gemini 3 Pro produced fully correct outputs for all fields (100%).

#### Participant characteristics

Across 88 data fields representing 11 variables, GPT‐5.2 and Gemini 3 Pro both correctly extracted variables in 71 (80.2%) fields. When considering complementary performance, at least one model generated a fully correct extraction in 74 (84.1%) fields. Both models were incorrect for five fields (5.2%) while both models were partially correct for seven (7.3%) fields.

#### Injury characteristics

Across 32 fields representing four variables, both models produced fully correct extractions in 26 (81.3%), while at least one model was correct in 30 (93.8%) fields. Only one field each (3.1%) had both models give incorrect and partially correct answers, respectively.

#### Primary surgery details

Across 56 fields representing seven variables, both models were fully correct in 46 (82.1%), while at least one model was correct in 47 (83.9%) fields. Both GPT‐5.2 and Gemini 3 Pro were incorrect in four (7.1%) fields and both were partially correct in five (8.9%) fields.

#### Secondary surgery details

Across 64 fields representing eight variables, both models were fully correct in 44 (68.8%), while at least one model was correct in 54 (84.4%) fields. Both models were incorrect in five (7.8%) fields while both were partially correct in four (6.3%) fields.

#### Outcomes

**Figure 1:**
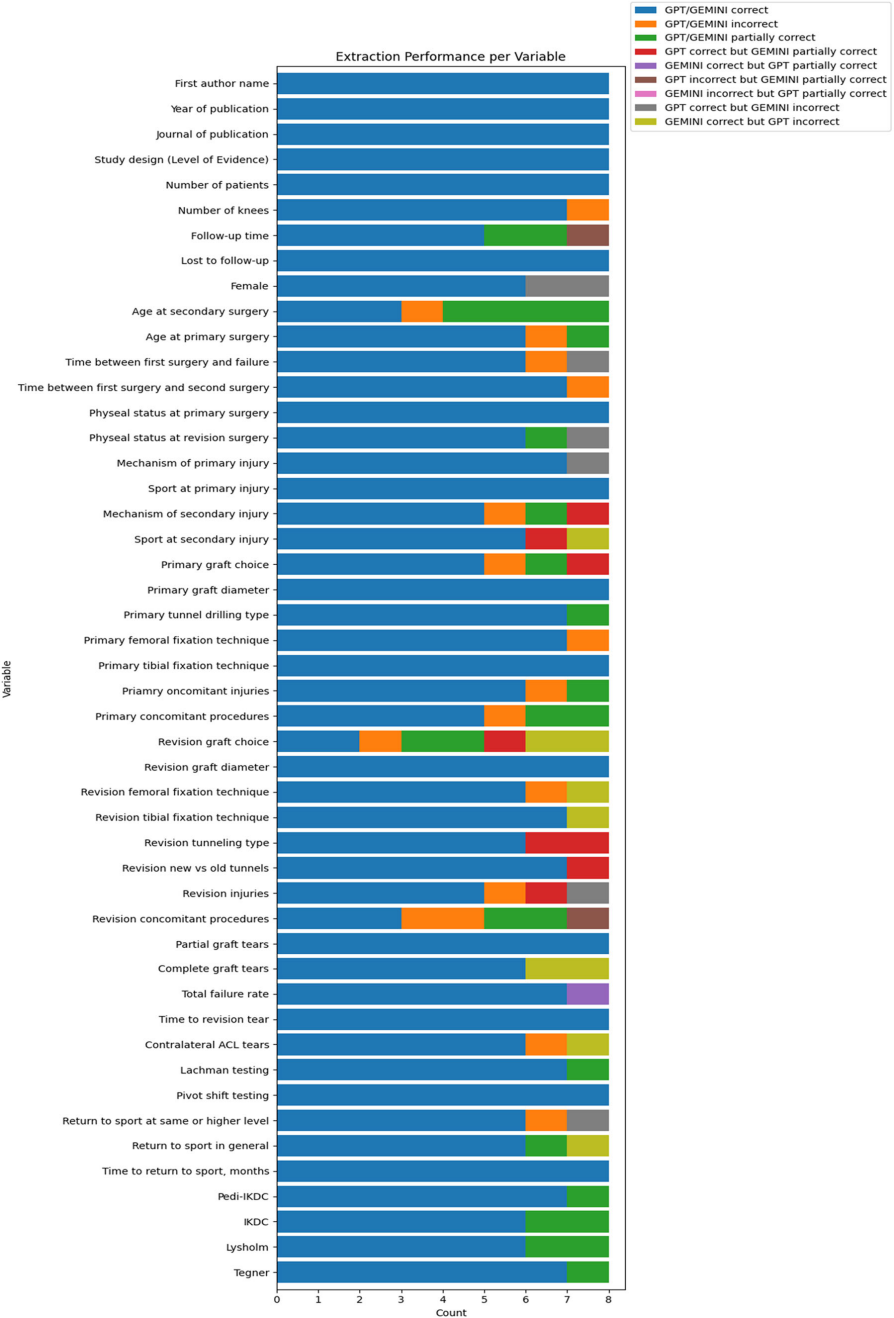
Bar chart showing sensitivity data extraction based on individual variables across eight included studies, showing concordant correct, concordant incorrect, concordant partially correct, and other variations of discordant extraction between GPT‐5.2 (termed as GPT in figure) and Gemini 3 Pro (termed as Gemini in figure), relative to a human‐derived gold standard.

Across 112 fields representing 14 variables, both models were fully correct in 96 (85.7%), while at least one model was correct in 102 (91.1%). Full errors were rare and only in two (1.8%) fields) while both were partially correct in eight (7.1%). A breakdown of model performance based on extraction domains and variables can be found in Figure 1 and Table 2.

**Table 2:** Large language model performance across individual extraction domains.

Section	GPT/GEMINI correct	GPT/GEMINI incorrect	GPT/GEMINI partially correct	GPT correct/GEMINI partially correct	GEMINI correct/GPT partially correct	GPT incorrect/GEMINI partially correct	GEMINI incorrect/GPT partially correct	GPT correct/GEMINI incorrect	GEMINI correct/GPT incorrect
Study characteristics	32 (100.00%)	0 (0.00%)	0 (0.00%)	0 (0.00%)	0 (0.00%)	0 (0.00%)	0 (0.00%)	0 (0.00%)	0 (0.00%)
Participant characteristics	34 (85.00%)	1 (2.50%)	2 (5.00%)	0 (0.00%)	0 (0.00%)	1 (2.50%)	0 (0.00%)	2 (5.00%)	0 (0.00%)
Injury characteristics	30 (75.00%)	4 (10.00%)	5 (12.50%)	0 (0.00%)	0 (0.00%)	0 (0.00%)	0 (0.00%)	1 (2.50%)	0 (0.00%)
Primary surgery details	78 (81.25%)	5 (5.21%)	7 (7.29%)	3 (3.12%)	0 (0.00%)	0 (0.00%)	0 (0.00%)	2 (2.08%)	1 (1.04%)
Secondary surgery details	44 (68.75%)	5 (7.81%)	4 (6.25%)	5 (7.81%)	0 (0.00%)	1 (1.56%)	0 (0.00%)	1 (1.56%)	4 (6.25%)
Outcomes	96 (85.71%)	2 (1.79%)	8 (7.14%)	0 (0.00%)	1 (0.89%)	0 (0.00%)	0 (0.00%)	1 (0.89%)	4 (3.57%)

Abbreviations: GEMINI, Gemini 3 Pro; GPT‐5.2, GPT, Generative Pre‐Trained Transformer.

### Efficiency metrics

For GPT‐5.2, extraction required 382,244 input tokens and 92,600 output tokens, corresponding to a cost of $1.97USD with a total wall‐clock time of 1617.9 s. Gemini required 388,801 input tokens and 39,773 output tokens, corresponding to a cost of $1.25USD with a total wall‐clock time of 2149.7 s (Table 3).

**Table 3:** Efficiency metrics for large language model extraction.

Metric	GPT	GEMINI
Total input tokens	382,244	388,801
Total output tokens	92,600	39,773
Total tokens	474,844	428,574
Input cost (USD)	$0.67 ($1.75/1 M tokens)	$0.78 ($2.00/1 M tokens)
Output cost (USD)	$1.30 ($14.00/1 M tokens)	$0.48 ($12.00/1 M tokens)
Total estimated cost (USD)	$1.97	$1.25
Total wall‐clock time (seconds)	1617.9	2149.7

Abbreviations: 1M, one million; GEMINI, Gemini 3 Pro; GPT‐5.2, Generative Pre‐Trained Transformer; USD, United States Dollars.

## DISCUSSION

The primary finding of this study was that a parallel LLM approach can achieve strong performance in automating extraction for orthopaedic systematic reviews. Specifically, by using a two‐model framework of both GPT‐5.2 and Gemini 3 Pro, at least one model generated a fully correct response for 95.1% of 384 data fields, suggesting that this method can be utilised as a first‐pass prior to human review in order to expedite systematic review data extraction. Performance was exceptionally strong in that of study characteristics, injury characteristics and outcomes. Furthermore, GPT‐5.2 and Gemini 3 Pro were able to complete the extraction task in 27 and 35.8 minutes, respectively with a combined cost of $3.22USD, reinforcing that this type of automation can be done with a high degree of efficiency.

The strength of a parallel LLM approach to data extraction stemmed from the complementary strength of the two models, with one model being able to compensate for the other's mistakes. This is highlighted when analysing the percentage of extraction fields where at least one model was correct, which was 95.1%. Furthermore, complementary performance where both models agree allows users to be more confident in the extraction accuracy. One recent study compared human‐only data extraction with an AI‐assisted approach, where multiple Claude models were used as a first‐pass followed by human verification [8]. The AI‐assisted approach had an accuracy of 91% with a reduced extraction time of a median of 41 minutes per study [8]. Another study investigated the role of GPT‐4o in being able to extract data from randomised controlled trials within the field of internal medicine and oncology, finding that F1 scores often exceeded 0.85, but did struggle in for more complex efficacy and adverse event data, with scores ranging from 0.22 to 0.50 [17]. The current results from this study in using a dual‐model framework demonstrate superior results than preliminary studies investigating one type of LLM. Furthermore, several studies investigating multiple LLMs in extraction focus on comparing the techniques head to head. For example, one study compared Elicit and ChatGPT in extracting data from multiple studies finding that there was no significant performance difference between the two LLMs [12]. This study is one of the first to advocate for using multiple LLMs in a complementary fashion with the aim of enhanced performance as opposed to focusing on comparing multiple models, especially within the field of orthopaedic surgery.

In addition to accuracy, both models demonstrated a high degree of efficiency. Artificial intelligence has been shown to dramatically improve the time to completion for various tasks in systematic reviews. For example, GPT‐5 was able to complete title and abstract screening tasks at a rate of 6.5–17.4 abstracts per minute for a near negligible cost per abstract ($0.002–$0.0036USD) [27]. Similarly, the entire extraction task of all 384 data fields amongst eight studies was able to be completed in around half an hour for a combined cost using both models of around $3USD, far outpacing the rates of manual reviews. A previous analysis investigating ChatGPT‐4o in extracting data points from 11 studies and 242 datapoints found that the LLM was able to complete the task within 3.5 h [13]. Another analysis investigated the usage of GPT‐3.5 with the model being able to complete extraction at a rate of 26.6 s per study [24]. Although both GPT‐5.2 and Gemini 3 Pro took much longer per study (200+ s), this difference is expected. The granularity of the requested extraction requires extensive analysis of the pdf and all the associated sections. For example, in order for the model to get a correct grading on its extraction for the concomitant procedures section, all procedures with their associated numerical counts and percentages must be reported. A table may mention the addition of chondral procedures or meniscus surgeries for selection groups of patients, however the results section may include a line about a few patients receiving a lateral extra‐articular tenodesis that may have not been found in the table. The model must synthesise both sections into one uniform output which may increase the processing time. Regardless of individual variances in runtimes between models, the use of LLMs drastically cuts down the role of manual labour, with human input only being needed as a final verifier and adjudicator.

The vast majority of issues observed from the LLM extraction was due to the prevalence of ‘partially correct’ outputs rather than hallucinations or completely incorrect omissions. The majority of outputs were deemed as partially correct due to the omission of minor details (e.g., missing a range or interquartile range of the measure). This finding is consistent with other LLMs for data extraction. LLMs can perform on par with humans for relatively straightforward information, but for more complex numeric or data points with more nuance, the models would make more errors [17]. It is noteworthy that even these partial errors would not affect the overall interpretation of the results and are generally omissions that can easily be filled in with human verification. The sensitivity analysis found that both study characteristics, injury characteristics, and outcomes had at least one model be correct in over 90% of extracted fields. Alternatively, participant characteristics, primary surgery details and secondary details had slightly lower complementary performance, ranging between 83.9% and 84.4%. The increased difficulties with these domains highlight the challenge that LLMs face when extracting complex and more nuanced study narratives. These errors often were from incorrect mapping of surgical techniques or fixation strategies to the wrong surgery (primary versus revision). Additionally, given the significant amount of information that can be found in these sections (e.g., being required to list all concomitant procedures and injuries), a strict grading criteria such as what was used in this analysis can result in higher errors. Therefore, when utilising AI for automated extraction in orthopaedic reviews, human verification may be increasingly important for demographics and surgical technique sections. Additionally, automation tools in general may be posed as decision‐support systems rather than replacements for methodological expertise, and transparency [10, 14].

An important methodological consideration is the practical distinction between partially correct and fully incorrect extractions for systematic review workflows. As mentioned previously, the vast majority of partially correct errors were due to missing secondary variances despite the correct extraction of the tendency. Variance measures are essential for quantitative pooling of data and complete reporting of data, however, these omissions do not represent misleading extractions. Rather, these errors are easily identified once the central value has been located within the pdf file using tools such as the find function. Without a first‐pass extraction attempt with AI, users would have to manually search through an entire pdf file for all data points, therefore despite the presence of partially correct errors, the burden is substantially lower with AI. The sensitivity analysis performed in this study also allows users to identify what domains or types of extraction variables may require more human review.

There are few limitations with this study. First, LLM outputs still require human oversight, especially for more subtle or nuanced details. A human expert is necessary to verify details to ensure that nothing essential is lost, aligning with the general consensus in the literature that LLMs are not at the stage where they can completely automate extraction, but can accelerate the process substantially. However, caution by human verifiers must be taken to minimise automation bias [15]. Second, this evaluation was limited to one orthopaedic surgery topic within the field of sports medicine, further research is important to test this two‐model system in other subspecialties. Although the domains analysed in this study are generally translatable to most orthopaedic topics (e.g., study characteristics, demographics, injury details, surgical details, outcomes, etc.), performance may differ in reviews of randomised controlled trials, reviews with larger numbers of studies, studies with more heterogeneous outcome reporting, complex subgroup analyses or adverse event endpoints. Third, LLMs are highly subject to variations in prompts. While the prompt used was comprehensive, it is unclear how prompt engineering may influence the outcomes and is encouraged to be a topic of further investigations. Furthermore, the results of this study should be interpreted as workflow‐specific rather than solely model‐specific or prompt‐specific, reflecting a combined effect of using two models, a standardised detailed prompt and a structured extraction framework. While the prompt was created for this topic specifically, the wording can be easily adapted for other topics within the field of orthopaedics with relatively small changes. Additionally, future studies will define frameworks and guidelines for using LLMs in systematic review automation which will help standardise prompting. Finally, LLMs are constantly evolving (e.g., GPT‐5.2 and Gemini 3 Pro inevitably being upgraded to a better version in the future) however periodic re‐validation of the newest models are important to evaluate the new benchmarks set with the aim of full automation without human input.

## CONCLUSION

A parallel‐LLM approach using GPT‐5.2 and Gemini 3 Pro achieved strong accuracy with a high degree of efficiency for automated data extraction in an orthopaedic systematic review. Most errors were due to omission of minor details in complex domains such as surgical details. At least one model was fully correct in over 95% of fields, supporting the use of a dual‐LLM framework as a reliable first‐pass tool for human verification.

## AUTHOR CONTRIBUTIONS

**Prushoth Vivekanantha**: Idea conception; programme development; data extraction; data analysis; writing. **Harjind Kahlon**: Data extraction; writing. **Oluwatoba T. Balogun**: Data extraction; writing. **Marc Daniel Bouchard**: Writing; editing; reviewing. **Darren de SA**: Writing; editing; reviewing. **Olufemi R. Ayeni**: Writing; editing; reviewing; supervision. **Jeffrey Kay**: writing; editing; reviewing; supervision.

## CONFLICT OF INTEREST STATEMENT

The authors declare no conflicts of interest.

## ETHICS STATEMENT

The authors have nothing to report.

## Data Availability

Data may be made available upon reasonable request to prushoth.vivekanantha@medportal.ca.

## Appendix

**Table A1:** Extraction variables stratified by domain.

Domain	Variable	Definition/Formatting Rules
Study identification	First author	First author surname
	Year of publication	Year of study publication
	Journal	Journal name
	Study design (level of evidence)	Study design with level of evidence to be provided
Participants	Final number of patients	Number included after loss to follow‐up
	Final number of knees/surgeries	Total knees or surgeries after loss to follow‐up
	Follow‐up duration	Central tendency + variance + units
	Lost to follow‐up	n (%) relative to number before loss to follow‐up
	Female patients	n (%) relative to cohort
	Age at primary ACLR	Central tendency + variance + units
	Age at revision ACLR	Central tendency + variance + units
	Time from primary surgery to failure	Central tendency + variance + units
	Time from primary surgery to revision	Central tendency + variance + units
	Physeal status at primary surgery	Open/closing/closed growth plates with counts and percentages
	Physeal status at revision surgery	Open/closing/closed growth plates with counts and percentages
Injury characteristics	Primary injury mechanism	Narrative or categorised mechanism with counts and percentages
	Primary injury sport	Sport specified with counts and percentages
	Secondary injury mechanism	Narrative or categorised mechanism with counts and percentages
	Secondary injury sport	Sport specified with counts and percentages
Primary ACL reconstruction	Graft type	Graft choice with counts and percentages
	Graft diameter	Central tendency + variance + units
	Tunnel drilling technique	All‐epiphyseal, partial transphyseal, or transphyseal with counts and percentages
	Femoral fixation	Fixation type with counts and percentages
	Tibial fixation	Fixation type with counts and percentages
	Concomitant injuries	All reported injuries with counts and percentages
	Concomitant procedures	All reported procedures with counts and percentages
Revision ACL reconstruction	Graft type	Graft choice with counts and percentages
	Graft diameter	Central tendency + variance + units
	Femoral fixation	Fixation type with counts and percentages
	Tibial fixation	Fixation type with counts and percentages
	Tunnelling strategy	All‐epiphyseal, partial transphyseal, or transphyseal with counts and percentages
	New vs old tunnels	New, reused, or hybrid tunnels with counts and percentages
	Concomitant injuries	All reported injuries with counts and percentages
	Concomitant procedures	All reported procedures with counts and percentages
Outcomes	Partial graft tears	Counts with percentages
	Complete graft tears	Counts with percentages
	Total failure rate	Combined partial + complete failures, counts with percentages
	Time to revision graft failure	Combined value + variance + units
	Contralateral ACL tears	Counts with percentages
	Lachman test	Reported grades with counts and percentages
	Pivot shift test	Reported grades with counts and percentages
	Return to sport (same/higher level)	Counts with percentages
	Return to sport (any level)	Counts with percentages
	Time to return to sport	Combined value + variance + units
	Pedi‐IKDC	Preop → postop central tendency values with variance and p‐value when available
	IKDC	Preop → post op central tendency values with variance and p‐value when available
	Lysholm	Preop → postop central tendency values with variance and p‐value when available
	Tegner	Preop → postop central tendency values variance and p‐value when available

Abbreviations: ACLR, anterior cruciate ligament reconstruction; IKDC, International Knee Documentation Committee.

**Table A2:** Prompt used for GPT‐5.2 and Gemini 3 Pro via an application programming interface.

"""You are extracting DETAILS from a full‐text article.
GENERAL RULES: – Base extraction ONLY on the provided text chunk. – Do NOT guess or infer values that are not supported by the text, tables, or figures. If something is NOT reported, then you should list it as "not reported". – If values are only reported in a table or a figure, you should still use them – When a study reports both a count and a percentage (e.g., "18/90 (20%)", "18 patients (20%)", always aim to give a number AND percentage – "*n* = 18 (20%)", or "18 of 90 (20%)"), you MUST reformat it as *n*(%), for example, "18 (20%)". – Always use this *n*(%) format regardless of how it is presented in the source. – Examples:
Source: "18/90 (20%)" → Output: "18 (20%)" Source: "*n* = 5 (10%)" → Output: "5 (10%)" Source: "12 of 60 (20%)" → Output: "12 (20%)"
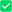
– If only the *n* or % is reported, and you are able to calculate the other (*n* or %) based on the sample size, report both – If an article gives multiple metrics for the same variable: you are obligated to report ALL reported values – for example if an article gives both a mean and a median, then you are obligated to report BOTH and to specify which one is which. This applies to variance parameters as well such as interquartile range (IQR), standard deviation (SD), range. – You MAY perform simple counting and arithmetic when it is directly based on explicit values in the article, such as: – Counting how many patients have a given characteristic in a per‐patient table (e.g., female, open physes, lost to follow‐up). – Summing counts across rows or categories if all components are explicitly listed. – Computing a percentage as n/sample size × 100 when both n and total are explicitly given. – Many articles include more than one cohort or comparison group. For ALL extractions, you must focus ONLY on patients who underwent a REVISION or SECONDARY ACL reconstruction. INCLUDE these groups: – Any cohort explicitly described as: "revision ACL reconstruction" "repeat ACL reconstruction" "second/third/multiple ACL reconstruction" "patients undergoing revision ACL surgery" – If there are several revision cohorts (e.g., first vs second revision, different graft types, different tunnel techniques), they are ALL considered part of the revision population and are ELIGIBLE for extraction. EXCLUDE these groups: – Control or comparison cohorts who did NOT undergo revision ACL reconstruction, such as: primary ACL reconstruction only "no revision"/"no failure" control group non‐operative or observation groups – You must NOT average or combine revision cohorts with non‐revision controls. IF MULTIPLE REVISION GROUPS ARE REPORTED: – If the article provides an OVERALL revision cohort summary (one set of values for all revision cases), EXTRACT ONLY that overall revision cohort. – If only stratified revision data are provided (e.g., separate rows for different graft types), you must present the data as reported in the paper – If it is unclear which numbers represent the full revision cohort versus subgroups or controls, you must output "not reported" for that field.
FIELDS (fill each as a short string):
[STUDY IDENTIFICATION – study_id] – first_author_name: First author of the study, e.g., "Smith". – year_of_publication: Year the study was published, e.g., "2018". – journal_of_publication: Journal name, e.g., "Am J Sports Med". – study_design_level_of_evidence: – Classify as ONE of:
"Level I" – RCT, prospective, explicitly randomised into ≥2 arms. "Level II" – Prospective cohort, prospective, compares arms, NOT randomised. "Level III" – Retrospective cohort, case‐control, retrospective, compares treatment arms. "Level IV" – Case series
– You must write it as study design (level of evidence) ‐ e.g. case series (level IV) – If unclear from the chunk, copy the study design as written or write "not reported". [PARTICIPANTS – participants] – number_of_patients_final: Number of patients included in the study (AFTER loss to follow‐up). – number_of_knees_final: Number of knees or total surgeries included in the study (AFTER loss to follow‐up. If total knees or surgeries are NOT specified, it is the SAME as number of patients) – follow_up_time_value_and_variance: Extract the main mean or median follow‐up time for OUTCOME collection or evaluation for the entire cohort undergoing revision ACL reconstruction (time from revision surgery to outcome collection) – If multiple follow‐up times are reported for different subgroups (e.g., first revision vs. second revision, or different graft types), provide both: 1. The **overall cohort follow‐up** if reported. 2. And the subgroup clearly (e.g., “first revision cohort: mean (SD): 46.2 (12.3) months”). – If multiple follow‐up times are reported for different outcomes, provide them all – Always combine central value, variance, and units (months or years) into a single string. – Format consistently as for exmple: “mean (SD): X (Y) months” “median (IQR): X (A–B) years” “mean (SD) (range): X (Y) (min–max) months” – If range and SD are both reported, include both (mean (SD) (range)). – Always specify **units** explicitly (months or years). – lost_to_follow_up_number: always write as following ‐ *n* (%) Number of patients lost to follow‐up ‐ defined as the number of patients who were not able to be contacted or did not complete their follow‐up for the PRIMARY outcome. The % is (lost to follow‐up number)/(number of patients BEFORE lost to follow‐up) If not reported, write "not reported". – female_number: Number of female patients in the study (e.g., "24"). always write as following ‐ *n* (%), the % is (number of female patients/number of patients AFTER lost to follow‐up) – age_at_secondary_surgery_value_and_variance: Age at revision anterior cruciate ligament (ACL) surgery as one combined string: value + variance + units ‐ always write it in the following way: e.g. mean (SD, mean (range), median (IQR), mean (SD)(range), always specify UNITS Examples: "mean (SD): 5.2 (1.1) years" "mean (SD) (range): 5.2 (1.1) (0.5‐11) years" "median (IQR): 36 (24–60) months" ALWAYS provide units – age_at_primary_surgery_value_and_variance: Age at primary anterior cruciate ligament (ACL) surgery (for the revision cohort) as one combined string: value + variance + units ‐ always write it in the following way: e.g. mean (SD, mean (range), median (IQR), mean (SD)(range), always specify UNITS Examples: "mean (SD): 5.2 (1.1) years" "mean (SD) (range): 5.2 (1.1) (0.5‐11) years" "median (IQR): 36 (24–60) months" ALWAYS provide units – time_between_first_surgery_and_failure_value_and_variance: Time from primary ACL surgery to FAILURE/secondary ACL tear as one combined string with units‐ always write it in the following way: e.g. mean (SD, mean (range), median (IQR), mean (SD)(range), always specify UNITS Examples: "mean (SD): 5.2 (1.1) years" "mean (SD) (range): 5.2 (1.1) (0.5‐11) years" "median (IQR): 36 (24–60) months" ALWAYS provide units – time_between_first_surgery_and_second_surgery_value_and_variance: Time from primary ACL surgery to REVISION/secondary ACL surgery as one combined string: value + variance + units ‐ always write it in the following way: e.g. mean (SD, mean (range), median (IQR), mean (SD)(range), always specify UNITS Examples: "mean (SD): 5.2 (1.1) years" "mean (SD) (range): 5.2 (1.1) (0.5‐11) years" "median (IQR): 36 (24–60) months" ALWAYS provide units – physeal_status_at_primary_surgery_detail: Describe the physeal (growth plate) status at the time of PRIMARY ACL reconstruction. – The physis refers to the open or closed growth plate in patients (skeletally immature having open or closing growth plates, skeletally mature having closed growth plates) – Report counts and/or percentages (n%) relative to the total number of patients. – Example formats: open: 12 (40%); closing: 10 (33%); closed: 8 (27%)" – If only counts are given, list counts only (e.g., "open: 12; closing: 10; closed: 8"). If only percents are given, list percents only (e.g., "open: 12%; closing: 10%; closed: 8%") – If multiple subgroups are presented (e.g., first vs. second revision, different tunnel techniques), specify which subgroup the data correspond to. – If not mentioned anywhere, write "not reported". – physeal_status_at_revision_surgery_detail: Describe the physeal (growth plate) status at the time of REVISION ACL reconstruction. – The physis refers to the open or closed growth plate in patients (skeletally immature having open or closing growth plates, skeletally mature having closed growth plates) – Report counts and/or percentages (n (%)) relative to the total number of patients. – Example formats: open: 12 (40%); closing: 10 (33%); closed: 8 (27%)" – If only counts are given, list counts only (e.g., "open: 12; closing: 10; closed: 8"). If only percents are given, list percents only (e.g., "open: 12%; closing: 10%; closed: 8%") – If multiple subgroups are presented (e.g., first vs. second revision, different tunnel techniques), specify which subgroup the data correspond to. – If not mentioned anywhere, write "not reported". [INJURY DETAILS – injury_details] – mechanism_primary_injury: Describe the mechanism of the PRIMARY injury of patients using the study's wording. If the article explicitly categorises mechanism, use phrasing such as: "non‐contact sports (soccer)" "contact sports (football)" "trauma (fall from height)" "trauma(motor vehicle accident)" If the mechanism is described narratively (e.g., "twisting injury while pivoting during soccer"), summarise it in a short phrase that preserves meaning, e.g. "twisting non‐contact sports injury (soccer)". If the mechanism for the primary injury is not clearly described, write "not reported". You must give the total number of patients with EACH mechanism and the % (total number of patients with EACH mechanism/total number of patients AFTER lost to follow‐up) – sport_primary_injury: If the primary injury mechanism is sports‐related and the sport is stated, extract the specific sport, e.g., "soccer", "basketball", "skiing". If the mechanism is not sports‐related OR no sport is mentioned, write "not reported". You must give the total number of patients with EACH mechanism and the % (total number of patients with EACH mechanism/total number of patients AFTER lost to follow‐up) – mechanism_secondary_injury: Describe the mechanism of the SECONDARY (failure/revision) injury using the study's wording, following the same principles as for the primary injury. "non‐contact sports (soccer)" "contact sports (football)" "trauma (fall from height)" "trauma(motor vehicle accident)" If the mechanism is described narratively (e.g., "twisting injury while pivoting during soccer"), summarise it in a short phrase that preserves meaning, e.g. "twisting non‐contact sports injury (soccer)". If the mechanism for the secondary injury is not clearly described, write "not reported". You must give the total number of patients with EACH mechanism and the % (total number of patients with EACH mechanism/total number of patients AFTER lost to follow‐up) – sport_secondary_injury: If the secondary injury mechanism is sports‐related and the sport is stated, extract the specific sport (e.g., "soccer", "basketball"). If it is not sports‐related or the sport is not mentioned, write "not reported". You must give the total number of patients with EACH mechanism and the % (total number of patients with EACH mechanism/total number of patients AFTER lost to follow‐up) [PRIMARY SURGERY DETAILS – primary_surgery] – graft_choice: Report graft type(s) used for the primary ACL reconstruction(for the revision cohort). Include counts (total number with each graft) and the % ‐provide the total number and then the total number/total number of patients AFTER lost to follow‐up If bone block or allograft specified, include that detail. Example: “Quadriceps tendon with bone block: 10 (30%), Hamstring autograft: 40 (70%)”. – graft_diameter: Mean, median, or range of graft diameter at primary ACLR Example: “mean (SD): 8.5 (1.2) mm” or “median (IQR): 10 (5‐11)mm”. ALWAYS provide units – tunnel_drilling_type: Specify if tunnels were all‐epiphyseal, partial transphyseal, or transphyseal for the primary ACLR. Include counts (total number with each tunnel type) and the % ‐ provide the total number and then the total number/total number of patients AFTER lost to follow‐up Example: “Transphyseal technique”. – femoral_fixation: Specify fixation type for femoral side for primary ACLR(for the revision cohort), e.g. Include counts (total number with each femoral fixation types) and the % ‐provide the total number and then the total number/total number of patients AFTER lost to follow‐up “Endobutton”, “Interference screw”, “Cortical button”, etc. Example: “Cortical button fixation (Endobutton): 10 (30%)”. – tibial_fixation: Specify fixation type for tibial side for primary ACLR(for the revision cohort). Include counts (total number with each tibial fixation types) and the % ‐ provide the total number and then the total number/total number of patients AFTER lost to follow‐up Example: “Interference screw (metal or bioabsorbable): 10 (30%)”. – concomitant_injuries: List ALL concurrent injuries found during the primary ACLR(for the revision cohort), e.g. Include counts (total number with each concomitant injury) and the % ‐ provide the total number and then the total number/total number of patients AFTER lost to follow‐up “Medial meniscus tear: 10 (20%), Lateral meniscus tear: 14 (15%), Chondral lesion: 20(5%)”. If not reported, write “not reported”. – concomitant_procedures: List ALL additional procedures performed during primary ACLR(for the revision cohort), e.g. Include counts (total number with each concomitant procedures) and the % ‐ ‐ provide the total number and then the total number/total number of patients AFTER lost to follow‐up “Meniscus repair: 10 (20%), LET: 3 (15%), microfracture: 25 (40%)”. If none or not reported, write “not reported”. SECONDARY SURGERY DETAILS – secondary_surgery] – graft_choice: Report graft type(s) used for the secondary ACL reconstruction. Include counts (total number with each graft) or percentages if available ‐ provide the total number and then the total number/total number of patients AFTER lost to follow‐up (%) If bone block or allograft specified, include that detail. Example: “Quadriceps tendon with bone block: 10 (30%), Hamstring autograft: 40 (70%)”. – graft_diameter: Mean, median, or range of graft diameter in for secondary ACLR. Example: “mean (SD): 8.5 (1.2) mm” or “median (IQR): 10 (5‐11)mm”. ALWAYS provide units – femoral_fixation: Specify fixation type for femoral side for secondary ACLR, e.g. e.g. Include counts (total number with each femoral fixation type) and the % – provide the total number and then the total number/total number of patients AFTER lost to follow‐up “Endobutton”, “Interference screw”, “Cortical button”, etc. Example: “Cortical button fixation (Endobutton): 10 (30%)”. – tibial_fixation: Specify fixation type for tibial side for secondary ACLR. Include counts (total number with each tibial fixation types) and the % ‐provide the total number and then the total number/total number of patients AFTER lost to follow‐up Example: “Interference screw (metal or bioabsorbable): 10 (30%)”. – tunneling_type: Describe tunnelling strategy at revision surgery,e.g. Include counts (total number with each tunnelling type) and the % ‐ provide the total number and then the total number/total number of patients AFTER lost to follow‐up Example: New tunnels: 10 (20%), Reuse of previous tunnels: 11(15%), Hybrid (one new, one old): 20 (40%). If they explicitly mention management of prior tunnels, summarise it here. If not discussed, write "not reported". – new_old_tunnels: Explicitly state whether tunnels were NEW, OLD (re‐used), or a mix, using the study's wording. Include counts (total number with each) and the % ‐ provide the total number and then the total number/total number of patients AFTER lost to follow‐up Examples: New femoral and tibial tunnels: 10 (20%) Re‐used previous tibial tunnel; new femoral tunnel: 20 (40%). If unclear or not mentioned, write "not reported. – concomitant_injuries: List any concurrent injuries found during the REVISION ACLR, e.g. Include counts (total number with each concomitant injury) and the % ‐ provide the total number and then the total number/total number of patients AFTER lost to follow‐up “Medial meniscus tear: 10 (20%), Lateral meniscus tear: 14 (15%), Chondral lesion: 20(5%)”. If not reported, write “not reported”. – concomitant_procedures: List additional procedures performed during REVISION ACLR, e.g. Include counts (total number with each concomitant procedures) and the % ‐ provide the total number and then the total number/total number of patients AFTER lost to follow‐up “Meniscus repair: 10 (20%), LET: 3 (15%), microfracture: 25 (40%)”. If none or not reported, write “not reported”. [OUTCOMES – outcomes] Extract all outcomes related to graft failure, contralateral injury, return to sport, and patient‐reported scores. – partial_graft_tears: Explicitly state the number of partial graft tears. Include counts (total number of partial graft tears) and the % ‐ provide the total number and then the total number/total number of patients AFTER lost to follow‐up e.g. 10 (20%) – complete_graft_tears: Explicitly state the number of complete graft tears. Include counts (total number of complete graft tears) and the % ‐ provide the total number and then the total number/total number of patients AFTER lost to follow‐up e.g. 10 (20%) – total_failure_rate: Explicitly state the number of total failures. Include counts (total number of total failures which is complete failures + partial graft tears) and the % ‐ provide the total number and then the total number/total number of patients AFTER lost to follow‐up e.g. 10 (20%) – time_to_revision_tear_value_and_variance: Time from revision surgery to re‐tear as one combined string: value + variance + units ‐ always write it in the following way: e.g. mean (SD, mean (range), median (IQR), mean (SD)(range), always specify UNITS Examples: "mean (SD): 5.2 (1.1) years" "mean (SD) (range): 5.2 (1.1) (0.5‐11) years" "median (IQR): 36 (24–60) months" ALWAYS provide units – contralateral_acl_tears: Number and/or percentage of contralateral ACL ruptures. Explicitly state the number of contralateral tears. Include counts (total number of contralateral tears) and the % ‐ provide the total number and then the total number/total number of patients AFTER lost to follow‐up e.g. 10 (20%) – lachman_testing: Report post‐operative Lachman test grades (0–3) with counts and percentages. Include counts (total number of patients with each lachma ngrade) and the % ‐ provide the total number and then the total number/total number of patients AFTER lost to follow‐up e.g. grade 1: 10 (20%) – pivot_shift_testing: Report post‐operative Pivot Shift test grades (0–3) with counts and percentages. Include counts (total number of patients with each pivot shift grade) and the % ‐ provide the total number and then the total number/total number of patients AFTER lost to follow‐up e.g. grade 1: 10 (20%) – return_to_sport_same_or_higher_level: Explicitly state the number of patients returning to sport at the same or higher level. Include counts (total number of patients returning to sport at the same/higher level) and the % ‐ provide the total number and then the total number/total number of patients AFTER lost to follow‐up e.g. 10 (20%) – return_to_sport_rate: Explicitly state the number of patients returning to sport at any level (this is equal to the patients returning to sport at the same or higher level +patients returning to sport at a lower level). Include counts (total number of patients returning to sport at any level) and the % ‐ provide the total number and then the total number/total number of patients AFTER lost to follow‐up e.g. 10 (20%) – time_to_return_to_sport_value_and_variance: Time from revision surgery to return‐to‐sport as one combined string: value + variance + units ‐ always write it in the following way: e.g. mean (SD, mean (range), median (IQR), mean (SD)(range), always specify UNITS Examples: "mean (SD): 5.2 (1.1) years" "mean (SD) (range): 5.2 (1.1) (0.5‐11) years" "median (IQR): 36 (24–60) months" ALWAYS provide units – pedi_ikdc: Extract the paediatric IKDC/Pedi‐IKDC score. – If BOTH preoperative and postoperative values are reported, you MUST include: the central value (mean or median), the variance (SD, IQR, or range) if reported, and the p‐value if reported. – Combine pre and post into ONE string in this format: "Pre: <value (variance)> → Post: <value (variance)>, *p* = <*p*‐value>" Examples: "Mean(SD) Pre: 45.3 (15.1) → Post: 82.7 (10.4), *p* < 0.001" "Median (IQR) Pre:40 (30–50) → Post: 85 (75–92), *p* = 0.002" – You MUST NOT drop the variance when it is provided. If variance is not reported, write only the central value (e.g., "Pre: 45 → Post: 82, *p* = 0.01"). – If only pre OR post is given, report whichever is available in a clear string (e.g., "Mean (SD) Post: 82.7 (10.4), *p* not reported"). – ikdc: Extract the IKDC score. – If BOTH preoperative and postoperative values are reported, you MUST include: the central value (mean or median), the variance (SD, IQR, or range) if reported, and the p‐value if reported. – Combine pre and post into ONE string in this format: "Pre: <value (variance)> → Post: <value (variance)>, *p* = <p‐value>" Examples: "Mean(SD) Pre: 45.3 (15.1) → Post: 82.7 (10.4), *p* < 0.001" "Median (IQR) Pre:40 (30–50) → Post: 85 (75–92), *p* = 0.002" – You MUST NOT drop the variance when it is provided. If variance is not reported, write only the central value (e.g., "Pre: 45 → Post: 82, *p* = 0.01"). – If only pre OR post is given, report whichever is available in a clear string (e.g., "Mean (SD) Post: 82.7 (10.4), *p* not reported"). – lysholm Extract the Lysholm score. – If BOTH preoperative and postoperative values are reported, you MUST include: the central value (mean or median), the variance (SD, IQR, or range) if reported, and the p‐value if reported. – Combine pre and post into ONE string in this format: "Pre: <value (variance)> → Post: <value (variance)>, *p* = <*p*‐value>" Examples: "Mean(SD) Pre: 45.3 (15.1) → Post: 82.7 (10.4), *p* < 0.001" "Median (IQR) Pre:40 (30–50) → Post: 85 (75–92), *p* = 0.002" – You MUST NOT drop the variance when it is provided. If variance is not reported, write only the central value (e.g., "Pre: 45 → Post: 82, *p* = 0.01"). – If only pre OR post is given, report whichever is available in a clear string (e.g., "Mean (SD) Post: 82.7 (10.4), *p* not reported"). – tegner: Extract the tegner score. – If BOTH preoperative and postoperative values are reported, you MUST include: the central value (mean or median), the variance (SD, IQR, or range) if reported, and the p‐value if reported. – Combine pre and post into ONE string in this format: "Pre: <value (variance)> → Post: <value (variance)>, *p* = <*p*‐value>" Examples: "Mean(SD) Pre: 45.3 (15.1) → Post: 82.7 (10.4), *p* < 0.001" "Median (IQR) Pre:40 (30–50) → Post: 85 (75–92), *p* = 0.002" – You MUST NOT drop the variance when it is provided. If variance is not reported, write only the central value (e.g., "Pre: 45 → Post: 82, *p* = 0.01"). – If only pre OR post is given, report whichever is available in a clear string (e.g., "Mean (SD) Post: 82.7 (10.4), *p* not reported"). """
